# Scientific Comparison of Different Online Heart Rate Monitoring Systems

**DOI:** 10.1155/2011/631848

**Published:** 2011-07-07

**Authors:** Martin Schönfelder, Georg Hinterseher, Philipp Peter, Peter Spitzenpfeil

**Affiliations:** ^1^Institute of Public Health Research, Technical University of Munich, Connollystraße 32, 80809 Munich, Germany; ^2^Fachgebiet für Trainingswissenschaftliche Diagnostik, Technische Universität München, Connollystraße 32, 80809 Munich, Germany

## Abstract

Recent technical development focused on real-time heart rate monitoring instead of postexercise evaluation of recorded data. There are several systems on the market that allow direct and real-time monitoring of several individuals at the same time. The present study compared the systems of Polar, Acentas, Activio, and Suunto in a field test with twelve subjects regarding failure quota, operating distance, and ECG validity. Moreover, the installation and use of software and hardware were evaluated with a quality rating system. Chest belts were evaluated with a questionnaire, too. Overall the system of Acentas reached the best mark of all systems, but detailed results showed that every system has its advantages and disadvantages depending on using purpose, location, and weather. So this evaluation cannot recommend a single system but rather shows strength and weakness of all systems and additionally can be used for further system improvements.

## 1. Introduction

Over the last 25 years, heart rate monitors have been widely used in sports and sports science. Since the development of handy heart rate monitors (HRMs) with the size of a watch and appropriate flexible chest belts with electrodes to transmit signals wirelessly to the watch, measurement of heart rate (HR) has become an easily accessible and valuable tool for training and coaching purposes and conducting laboratory or field studies [1]. Validity and reliability have been shown to be high for these tools compared to ECG measurements for both HR and heart rate variability (HRV) [1–6]. Owed to its widespread use in physical activity not only for individuals but also for team sports, recent technical development focused on real-time monitoring instead of post-exercise evaluation of recorded data. This led to both software and hardware development of wireless online HR monitoring systems. Currently, several different systems are available on the market, of which four were tested in the present study: POLAR Team^2^ Pro, Acentas team monitoring system, Suunto Pro Team Pack and Activio Sport System. All systems allow direct and real-time monitoring of several individuals at the same time. Data are transmitted wirelessly to a receiver which is connected with a standard laptop with relevant software. However, a direct comparison of these systems and validity of recorded data are missing in literature. Therefore the main purpose of this study was to compare all systems in a field test against each other. Operating distance and validity against a commercially available ECG were evaluated as well as the installation and use of software and hardware of the above-mentioned systems.

## 2. Methods

### 2.1. Subjects

A total of 12 active people (11 men and 1 woman; age: 26.4 ± 3.2 yr; height: 177.9 ± 8.7 cm; mass: 78 ± 7 kg) participated in the study. Ten men of an amateur soccer team conducted an online field test during a real football match. One male subject participated for validity of HR monitoring systems against ECG whereas the female subject was only involved in operating distance measurement. Because of the low case number of the last two tests, data can only be considered as a pilot study for ECG validation and distance measurement.

### 2.2. Heart Rate Monitoring Systems

The following four commercially available systems were used for all tests: POLAR Team^2^ Pro (Polar Electro Oy, Kempele, Finland), Acentas team monitoring system (Acentas GmbH, Hörgertshausen, Germany), Suunto Pro Team Pack (Suunto Oy, Vantaa, Finland), and Activio Sport System (Activio AB, Stockholm, Sweden).

### 2.3. Quality Rating System

A quality rating system similar to rating systems used in trade journals was used to test electronic equipment and software. A valuable design was found in the journal “Connect” (http://www.connect.de/) which is specialised on the testing of mobile and electronic communication tools. For this purpose, the authors decide to subdivide the rating system into three main categories: measurement, software, and hardware. At this, the core area of the study focused on the adequate online measurement of the heart rate. Therefore, the rating system was set in favour of the measurement which was represented by 200 scores of maximal 500. The latter 300 were equally divided on quality of the hardware and the usability of the software. Detailed distribution into the several subcategories is depicted in Table 1. Subcategories were weighted subjectively by the authors in consideration of heart rate monitoring of team sports including the monitoring of 10 or more subjects in parallel. In addition, the following subcategories were rated once by an independent observer who was not familiar with any of the monitor systems to get a first impression: instruction manual, packing, chest belts (quality), and initial software handling. The remaining categories were judged once by an experienced person. With exception of wearing comfort and failing quota in field, all subcategories were judged and quantified by using a ten-stepped visual analogue scale which is known for pain assessment [7]. Visual analogue scaling was weighted by multiplying itself with factor *F*: (*F* = maximal score of subcategory/10). Parameters leading the judgement of the subcategories are described in Tables 2 and 4. 

Wearing comfort of the chest belts was evaluated via a short anonymous questionnaire which all 12 participants (see Section 2.1) had to complete directly after wearing. The questionnaire contained five questions about the handling and individual fitting of the chest belts. The questions one and two are positively formulated whereas questions three to five are negative (see Table 3). All questions had to be answered by an even scaled rating system including six categories. The six different categories range from “totally agree” = 5 points to “absolutely disagree” = 0 points. To compare positively and negatively formulated questions, the negative one was transformed before mean value was calculated. Maximum score for the whole questionnaire was 25; hence, each question was scored with maximal 5 points.

### 2.4. Measurements

The main field test was measurement of failure ratio during the half time of a real soccer match. All players apart from goal keeper were equipped with chest belts of the relevant system and measured during first half (= 45 min) of a league soccer game during four home matches in summer 2009. Temperature range was within 9°C, and weather conditions were similar (no precipitation) for all measurements. The receiver of the relevant system was always positioned three meters in projection of midline of the soccer ground. In order to improve transmission distance, receiver was positioned one meter above ground level. As data loss due to injury could not be excluded a priori, a mean failure ratio was determined only with transmitters sending valid data. Each deviation/loss of one percent of whole duration measurement of 45 min (= 2700 s) was subtracted with 10 scores, and a maximal score of 100 could be obtained. If, for example, a total loss time of all ten transmitters of a system was 270 s, a total score of 90 would be credited to this system. A failure ratio of 10 percent of whole duration measurement would be credited with a zero score.

Distance measurement was conducted on a uphill street with a constant slope and in far distance to high-voltage line or radio tower to reduce possible interference. Receiver antenna was placed on highest elevation of the hill one meter above ground, and unit was connected to a laptop (Toshiba Satellite Series; Toshiba Inc, NY, USA). The participants wearing the chest belts were equipped with a GPS device (Nokia N79, Nokia Group, Espoo, Finland) to measure distance. By moving backwards it was guaranteed that the chest belt transmitters were permanently faced to the receiver. Measurements were terminated when transmission signal was lost. Additionally, the participants were instructed to move arms to keep HR variable with time. Attempts were only started when both valid signals of GPS and HR were available and stopped when HR signal was lost. Tests were conducted twice, and mean value of both attempts was calculated and taken as maximal range. Maximal range distance was credited with 70 scores whereas the system with the absolute maximal covered range was taken as reference system and credited maximum score. Percentage deviation of distance of other systems was analogously deducted as percentage of scores. If, for example, reference system had 100 m range, it was credited 70 scores. If a second system had only 50 m range, it would be credited 50% of maximum score, hence 35.

Comparison against ECG was conducted in a laboratory by using ECG tool Vicardio ECP-6 standard (Energy-Lab Technologies, Hamburg, Germany). Measurement was conducted two minutes at rest with single-polar extremity drains. Mean HRs of both ECG and relevant system were compared. A maximum score of 30 could be obtained at this test. A deviation of 1 beat per minute (bpm) was deducted with two scores. Only complete accordance of both values was credited with maximum score.

## 3. Results

### 3.1. Comparison of Hardware

Results for hardware are summarized in Table 2. A plus (+) is a positive feature; a minus (−) is a negative one. If all systems were similar or identical, there is no separate classification. A plus/minus (±) indicates aspects which are both positive and negative. In context of the supplied hardware Acentas and Polar lead the field of monitor systems, both reaching 100 and 106 scores in sum for the hardware. The leading position of Polar depends on the storage capacity of the chest belt increasing data safety but also decreasing wearing performance which is indicated by the chest-belt questionnaire presented in Table 3. In case of wearing comfort, it has to be noted that both chest belts of Activio and Acentas were supplied by the identical manufacture. Both Polar and Acentas delivered high-quality receivers.

### 3.2. Comparison of Software

Results of software evaluation are summarized in Table 4. Again, similar to hardware evaluation, a plus (+) is a positive feature, and a minus (−) is a negative one. If all systems were similar or identical, there is no separate classification. A plus/minus (±) indicates aspects which are both positive and negative. Here, Activio offered the harmonious concept which led to the highest ranking. The installation procedure was easiest with the Acentas software, but Acentas did not focus on extensive statistical analysis. In this case, Polar as well as Activia provide a more developed software tool.

### 3.3. Measurement Results

As data of ECG comparison were only obtained with a single subject, they have to be regarded with caution. Further evaluation would be necessary to confirm or reject results. Data are represented against reference system. Acentas had 86 bpm average HR versus 88 of reference system. Suunto's deviation was 80 to 85 bpm, Activio's deviation was 81 to 85 bpm, and Polar's deviation was 82 to 85 bpm, respectively. Score distribution is depicted in Table 5. Concerning distance measurement, same caution has to be taken as only one subject's data were included in analysis. Nevertheless, Acentas gave reference with 349 m, followed by Activio with 141 m and Suunto with 107.5 m. It has to be noted that Activio's system was supposed to reach 300 m. Last but not least, Polar reached 102 m which corresponded to the manufacturer information for this system. Nevertheless, all other systems had a wider range of data acquisition, and hence score distribution was credited as follows: Acentas 70, Activio 28, Suunto 21, and Polar 20.

Regarding failure quota, average downtime was 148 s (0,6%) for Acentas, 286 s (1,0%) for Polar, and 552 s (2,0%) for Activio. The Suunto system stood out negatively, as a complete data loss was seen. Single belts were recognised from time to time, but no consistent data acquisition could be accomplished. A receiver problem might have caused this failure. Therefore, Suunto was not scored at all in this test. Similarly, five out of ten Polar chest belts had signal loss and could not be reactivated whereas Activio managed 10 out of 10 signals, and for the Acentas system 9 out of 10 were used for data evaluation as one person was injured during match play. The summarized scores and overall results are represented in Table 5.

## 4. Discussion

The purpose of the present study was to compare four commercially available online heart rate monitoring systems and especially their use in everyday situation in team sports. The system of Polar could score especially with a clear design, useable features of the software, and a convincing product quality. Only Polar uses WLAN for data transmission and also adds a mobile terminal in pocket format which can easily be used outdoor and with bad weather conditions. But the high energy consumption of WLAN transmission would terminate the battery capacities and additionally the duration of measurement. The system of Activio has a functional and useful software and the instruction manual was very detailed and easily comprehensible. The price, however, was very high compared with the other systems. Acentas could score with a huge operating distance, a valid heart rate signal compared with ECG, a low failure quota, and an easy handling. In contrast, the system of Suunto had a total breakdown during competition measurements and could not be reactivated. So this system was evaluated as the worst. The evaluation of the several chest belts revealed no important advantage of one manufacturer. Only the chest belt of Polar showed weaknesses regarding wearing comfort. Altogether, all systems cannot be used for online heart rate monitoring. Prospectively, it would be a great challenge to realise online monitoring for swimmers, possibly leading to new opportunities for the training regime. In this context the system of Polar and Suunto had the advantage that data are also recorded on the chest belt memory during water immersion and could be evaluated after exercise. 

Meanwhile, some of the tested heart rate monitoring systems are updated and upgraded with new functions, respectively. For example, Acentas included an offline memory in the chest belts and added a wrist monitor for direct heart rate analysis from up to twenty athletes by the coach.

In summary, Acentas system reached the best mark of all systems, but every system has its advantages and disadvantages depending on the using purpose, location, and weather. So this present comparative study cannot recommend a single system but rather shows strength and weakness of all systems and additionally can be used for further system improvements.

##  Disclosures

The investigators were responsible for the study design, data collection, data analysis, data interpretation, and submission of the paper for publication, independently of all funding sources. The authors declare that they have no financial conflict of interests to disclose with any of the companies named in this paper. There is no preferential order of companies in the paper.

## Figures and Tables

**Table 1:** 500-score quality rating system, overall rating system divided in three main categories: hardware, software, and measurements.

Main category	Subcategory	Item	Scores
Hardware	Instruction manual	understandability	10
	Packing	Quality	10
		Handling	15
	Chest belt	Storage	10
		Battery	10
		Quality	20
		Wearing comfort	25
	Receiver	Power consumption	5
		Quality	25
		Handling	15
		Connectivity	5

		Subtotal	**150**

	Installation		10
Software	Menue navigation		40
	Features		100

		Subtotal	**150**

	ECG deviation		30
Measurements	Distance		70
	Failure quota in field test		100

		Subtotal	**200**

Maximum overall score			**500**

**Table 2:** Notes on hardware comparison.

System	Acentas	Activio	Polar	Suunto
*Instruction manual * (language, comprehensibility, volume, usefulness)	(i) Short and compact but sufficient (+)	(i) Very detailed IM in English (+) (ii) Easily comprehensible (+)	(i) Short instruction in six languages (+) (ii) Laminated (+) (iii) Personal instruction by member of staff (+) (iv) Useful (+) (v) Only short hardware & installation instruction (−)	(i) No instruction manual (−)

*Packing * (quality, handling)	(i) Compact backpack (<1 kg) (+) (ii) Good accessibility & separation of hardware (+) (iii) Poor quality of packing system (−)	(i) Good arrangement of hardware (+) (ii) Heavy (+2 kg) (−) (iii) Hardware & software not fixed in packing (−) (iv) Poor quality of packing system (−)	(i) Carrying case (+) (ii) Good quality of packing system (+) (iii) Good arrangement of single system parts (+) (iv) Heavy (~3 kg) (−)	(i) Carrying case (+) (ii) Lightweight (~1kg) (+) (iii) Poor arrangement of hardware (−)

*Chest belt * (storage, battery, quality)	(i) Simple snap closing (+) (ii) Water resistant (+) (iii) Very good battery (+) (iv) No internal storage (−)	(i) Simple snap closing (+) (ii) Water resistant (+) (iii) Very good battery (+) (iv) No internal storage (no rating) (−)	(i) Bluetooth USB stick for storage unit (+) (ii) High storage capacity (+) (iii) Small, bulky snap closing (−) (iv) Poor battery (−)	(i) USB reader for storage unit (+) (ii) Two types of chest belt (±) (iii) Small snap closing (−) (iv) No driver for USB (& not found on homepage) (−) (v) Low storage capacity (−)

*Receiver * (power consumption, quality, handling, connectivity)	(i) Lightweight (~150 g) (+) (ii) Connection only via 150 cm USB cable (±) (iii) Water repellent (+) (iv) Power connection via USB from computer (+)	(i) Similar to Acentas system (±) (ii) Satisfactory quality (±) (iii) Power connection via USB from computer (+) (iv) Heavy weight antenna (~500 g) (−)	(i) Best quality (+) (ii) Power independent from computer (±) (iii) LAN and WLAN connection (+) (iv) Very heavy (~3 kg) (−) (v) WLAN connection only <50 m (−) (vi) WLAN energy consuming (−) (vii) Plug and power cable necessary (−)	(i) Lightweight (+) (ii) Good quality (+) (iii) Power connection via USB from computer (+) (iv) USB cable cannot be unplugged (−) (v) Control LED missing (−)

**Table 3:** Score from the chest-belt questionnaire. (0 = “totally agree”; 5 = “absolutely disagree”).

Statement	Acentas	Activio	Polar	Suunto
“The belt is easily fixed and locked”	3.8	3.4	2.8	2.9
“The belt is precisely and simply adapted to my chest girth”	3.1	3.2	3.2	2.6
“I consider the belt disturbing right after attaching it to the body”	0.9	1.0	0.9	1.1
“The belt hindered me during match play”	2.6	2.7	1.6	2.9
“The belt caused pain”	3.5	3.5	2.0	3.6

**Table 4:** Notes on software comparison.

System	Acentas	Activio	Polar	Suunto
*Installation*	(i) On USB stick (+) (ii) All drivers included (+)	(i) Easy installation (+) (ii) Driver problems (Win XP) (−)	(i) Installation by Polar personnel (+) (ii) Complicated installation process (−) (iii) Connectivity of LAN not existing (−) (iv) Permanent update function (−)	(i) No software included, download only on homepage (−) (ii) Same problem with drivers (−) (iii) Nevertheless, easily installed (+)

*Menu navigation * (intuitional, unproblematic)	(i) Automatic recognition of chest belts (+) (ii) Well-arranged & simple screen (+)	(i) Very good arrangement of functions & team function (+) (ii) Long-loading software (−) (iii) Manual recognition of chest belts (−)	(i) Long-loading software (−) (ii) Long recognition of each receiver unit (−) (iii) Complex software (−) (iv) Overloaded screen (−) (v) No automatically recording of data (−)	(i) Well-arranged & simple screen (+)

*Features * (user friendliness, help function, additional function, graphical representation of data, use and treatment of data)	(i) Simple personalisation of a subject (+) (ii) Complete pdf instruction manual (+) (iii) Good and easy graphical representation of data (+) (iv) Simple data recording & saving (+) (v) Good data export (+) (vi) No import of different systems' data possible (−)	(i) Simple and fast recognition of active chest belts (+) (ii) Good team function (+) (iii) Clear separation of single and team players (+) (iv) Help function did not work (−) (v) Good and easy graphical representation of data (+) (vi) Good data comparison & treatment (+) (vii) Simple data print, pdf creation and export (+) (viii) No import of different systems' data possible (−)	(i) Multifaced and user-friendly data creation (+) (ii) Comprehensive features (+) (iii) Extensive help function (+) (iv) Initial problems (−) (v) Good and easy graphical representation of data and saved sessions (+) (vi) Overloaded graphical presentation for several individuals (−) (vii) Email export (+) (viii) No import of different systems' data possible (−)	(i) Unlabelled belts, boxes (−) (ii) Time-consuming data input (−) (iii) Missing “team” function of monitor software (−) (iv) Poor search function (−) (v) Good help function (+) (vi) Good and easy graphical representation of data (+) (vii) Separate connection of every single player (−) (viii) Simple data recording & saving (+) (ix) No additional features (−) (x) No import of different systems' data possible (−)

**Table 5:** Summary of all scores.

Category	Maximum attainable score	Acentas	Activio	Polar	Suunto
Hardware	**150**				

*Instruction manual*	**10**	9	10	5	0
*Packing*	**25**				
Quality	10	7	5	10	7
Handling	15	14	5	5	6
*Chest belt*	**65**				
Storage	10	0	0	10	8
Battery	10	9	9	6	8
Quality	20	17	17	18	15
Wearing comfort	25	14	14	11	13
*Receiver*	**50**				
Power consumption	5	5	5	3	5
Quality	25	19	19	23	17
Handling	15	13	8	10	13
Connectivity	5	3	3	5	3

Software	**150**				

Installation	10	10	5	4	4
Menu navigation	40	35	38	30	35
Features	100	75	85	90	50

Measurements	**200**				

ECG-deviation	30	26	22	24	20
Distance	70	70	28	20	21
Failure quota	100	94	80	90	0

Overall score	500	420	353	364	225

Relative score (in %)		84	70	73	45

Price (in EUR)		ca. 3.500	ca. 6.000	ca. 3.500	ca. 3.000
